# Hepatobiliary Thyroid Hormone Deficiency Impacts Bile Acid Hydrophilicity and Aquaporins in Cholestatic C57BL/6J Mice

**DOI:** 10.3390/ijms232012355

**Published:** 2022-10-15

**Authors:** Irina Kube, Manuela Kowalczyk, Ute Hofmann, Ahmed Ghallab, Jan Georg Hengstler, Dagmar Führer, Denise Zwanziger

**Affiliations:** 1 Department of Endocrinology, Diabetes and Metabolism and Clinical Chemistry—Division of Laboratory Research, University of Duisburg-Essen, Hufelandstraße 55, 45147 Essen, Germany; 2 Dr. Margarete Fischer-Bosch Institute of Clinical Pharmacology and University of Tübingen, Auerbachstraße 112, 70376 Stuttgart, Germany; 3 Leibniz Research Centre for Working Environment and Human Factors, Department of Toxicology/Systems Toxicology, Ardeystraße 67, 44139 Dortmund, Germany; 4 Department of Forensic Medicine and Toxicology, Faculty of Veterinary Medicine, South Valley University, Qena 83523, Egypt

**Keywords:** gallstones, thyroid hormone deficiency, bile acids, aquaporins

## Abstract

Women are more prone to develop either hypothyroidism or cholesterol gallstones than men. However, a male predominance in cholesterol gallstones under hypothyroidism was reported. Recently, a novel pathogenic link between thyroid hormone (TH) deficiency and cholesterol gallstones has been described in male mice. Here, we investigate if TH deficiency impacts cholesterol gallstone formation in females by the same mechanism. Three-month-old C57BL/6J mice were randomly divided into a control, a TH deficient, a lithogenic, and a lithogenic + TH deficient group and diet-treated for two, four, and six weeks. Gallstone prevalence, liver function tests, bile composition, hepatic gene expression, and gallbladder aquaporin expression and localization were investigated. Cholesterol gallstones were observed in lithogenic + TH deficient but not lithogenic only female mice. Diminished hydrophilicity of primary bile acids due to decreased gene expression of hepatic detoxification phase II enzymes was observed. A sex-specific expression and localization of hepatobiliary aquaporins involved in transcellular water and glycerol permeability was observed under TH deficient and lithogenic conditions. TH deficiency promotes cholesterol gallstone formation in female C57BL/6J mice by the same mechanism as observed in males. However, cholesterol gallstone prevalence was lower in female than male C57BL/6J mice. Interestingly, the sex-specific expression and localization of hepatobiliary aquaporins could protect female C57BL/6J mice to cholestasis and could reduce biliary water transport in male C57BL/6J mice possibly contributing to the sex-dependent cholesterol gallstone prevalence under TH deficiency.

## 1. Introduction

Epidemiological studies suggest a link between hypothyroidism and cholesterol gallstone disease [1,2,3]. The hepatobiliary system is composed of the hepatocytes, gallbladder, and biliary tree and regulates the production, storage, and secretion of bile. Biliary supersaturation with cholesterol is thought to be the main cause of cholesterol gallstone formation [4]. In addition, altered gallstone (LITH) gene expression, impaired gallbladder motility associated with reduced bile flow, and intestinal factors can promote cholesterol gallstone disease [5]. In addition to the impact of thyroid hormone (TH) deficiency on hypercholesterolemia [6] and a diminished biliary secretion by reduced sphincter of Oddi motility [3], TH alters LITH gene expression [7,8,9] which could promote biliary supersaturation and cholesterol crystal precipitation. Interestingly, a female predominance in cholesterol gallstone disease has been observed under a systemic euthyroid state [10], whereas an independent association between hypothyroidism and cholesterol gallstone disease has been reported in the population-based Study of Health in Pomerania in men [2]. In mice, C57BL/6J females are less susceptible to cholesterol gallstone formation under a lithogenic diet than males [11]. In previous studies, an increased cholesterol gallstone prevalence under TH deficiency in male C57BL/6J mice could be observed [12]. Hepatic expression of genes involved in cholesterol, bile acid, and phosphatidylcholine synthesis and canalicular transport as well as biliary concentrations of cholesterol, bile acids, and phosphatidylcholine could not explain the increased cholesterol gallstone prevalence under TH deficiency. Biliary bile acids originate from newly synthesized primary and secondary bile acids returned to the liver via the enterohepatic circulation. Interestingly, TH deficiency decreased the hydrophilicity of primary but not secondary bile acids in the bile of male C57BL/6J mice. This decreased hydrophilicity of primary bile acids was accompanied by decreased gene expression of hepatic detoxification phase II enzymes and suggested a novel pathogenic link between TH deficiency and cholesterol gallstone formation. However, if TH deficiency affects cholesterol gallstone formation by the same mechanism in female mice and in how far TH deficiency contributes to sex-dependent cholesterol gallstone susceptibility has not been addressed so far. Here, we investigated the impact of TH deficiency in cholesterol gallstone disease using a mouse model of C57BL/6J mice under lithogenic conditions and modulation of the TH status. In this model, we show that TH deficiency leads to cholesterol gallstone formation by diminished bile acid hydrophilicity of lithogenic diet-supplemented female mice. Moreover, differences in hepatobiliary aquaporin expression and localization could impact sex-specific cholesterol gallstone prevalence under TH deficient condition.

## 2. Results

### 2.1. Hepatobiliary Histology and Function under Thyroid Hormone Deficient and Lithogenic Conditions in Female C57BL/6J Mice

**Figure 1:**
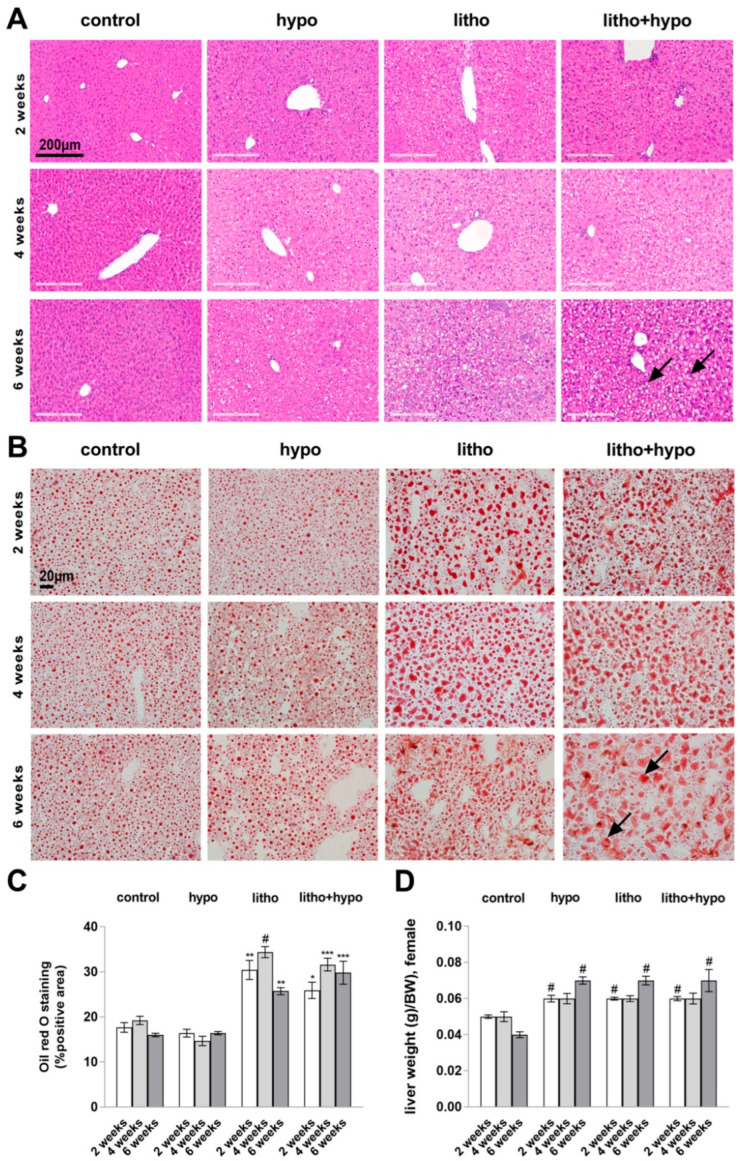
Increased hepatic lipid accumulation under lithogenic diet in female C57BL/6J mice. (**A**) Representative HE stainings reveal bright plasmatic cells (arrows) under litho and litho+hypo conditions indicating lipid accumulation (scale bar = 200 µm), n = 6–8/condition. (**B**) Increase in hepatic lipid content (arrows) in litho and litho+hypo mice determined by Oil Red O staining (scale bar = 20 µm). (**C**) Oil Red O staining quantification, n = 4/condition. (**D**) Increased wet liver weight (g/BW) under hypo, litho and litho+hypo conditions as compared to control, n = 6–8/condition. Data are represented as mean ± SEM, one-way ANOVA followed by Bonferroni post hoc analysis, * *p* < 0.05, ** *p* < 0.01, *** *p* < 0.001, # *p* < 0.0001.

Successful induction of TH deficiency was confirmed by analysis of total thyroxine (TT4) serum concentrations showing significantly reduced TT4 levels in TH deficient mice (hypo, litho+hypo) compared to controls (Supplementary Table S1). Hepatic thyroid state was assessed by mRNA expression analysis of TH-responsive genes *Dio1* and *Tbg*, respectively. TH deficiency (hypo, litho+hypo) reduced *Dio1* expression and increased *Tbg* expression (Supplementary Table S1). In addition, lithogenic diet (litho) increased TT4 serum concentration as compared to controls and reduced hepatic *Dio1* expression, but to a lesser extent as compared to hypo and litho+hypo conditions (Supplementary Table S1). TH deficiency and lithogenic diet increased bright plasmatic cells and wet liver weight (Figure 1A,D). In addition, lithogenic diet increased hepatic lipid content demonstrated by elevated hepatocyte lipid droplets formation (Figure 1B,C). Serum transaminase activities were slightly elevated under litho and litho+hypo conditions compared to controls (Supplementary Figure S1).

### 2.2. TH Deficiency Leads to Cholesterol Gallstone Formation in Lithogenic Diet Supplemented Female C57BL/6J Mice

**Figure 2:**
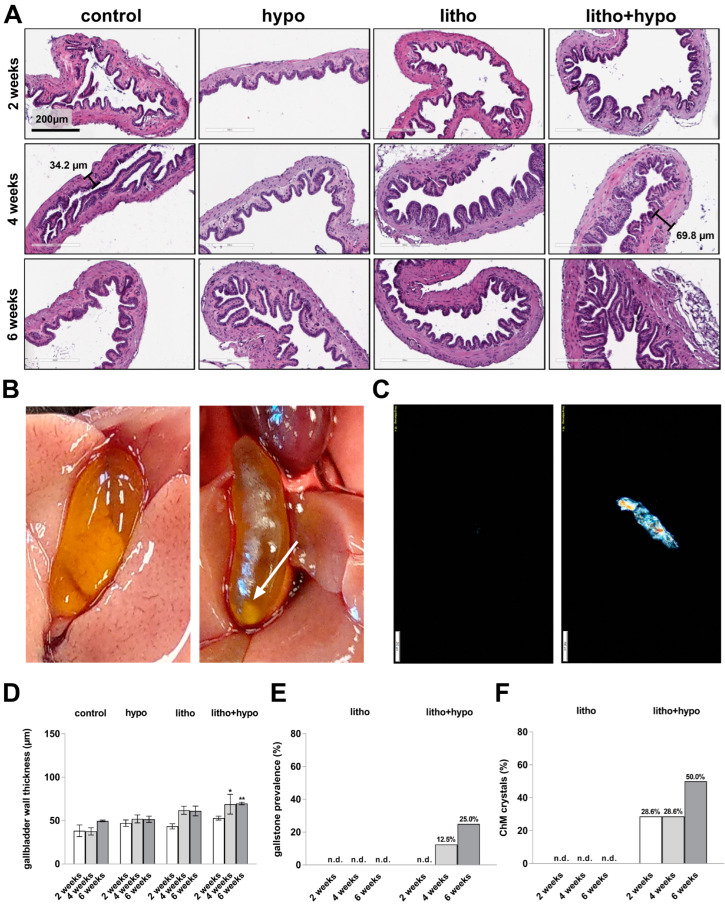
Thyroid hormone deficiency increases gallbladder wall thickness and leads to cholesterol gallstone and cholesterol monohydrate crystal formation in lithogenic diet-supplemented female C57BL/6J mice. (**A**) HE staining of gallbladder tissue shows increased gallbladder wall thickness under litho+hypo condition (example shown, 20× magnification, n = 3–4/condition). (**B**) Left: representative gallbladder without macroscopic gallstones. Right: representative gallbladder with macroscopic gallstones (white arrow), n = 6–8/condition. (**C**) Left: representative photomicrograph of biliary fluid without ChM crystals; Right: representative photomicrograph of biliary fluid with ChM crystals detected by light polarization microscopy, n = 4–8/condition. (**D**) Quantification of the gallbladder wall thickness by measuring six different positions per sample in Image J. Increased gallbladder wall thickness under litho+hypo condition, n = 3–4/condition. Data are represented as mean ± SEM, one-way ANOVA followed by Bonferroni post hoc analysis, * *p* < 0.05, ** *p* < 0.01. (**E**) Gallstone prevalence (%). After 4 and 6 weeks, TH deficiency leads to gallstone prevalence of lithogenic diet-supplemented mice (4 weeks: 0% vs. 12.5%; 6 weeks: 0% vs. 25%); n.d.: not detected. (**F**) Cholesterol monohydrate (ChM) crystal prevalence in bile (%). TH deficiency leads to biliary ChM crystal presence in mice under lithogenic diet (2 and 4 weeks: 0% vs. 28.6%; 6 weeks: 0% vs. 50.0%); n.d.: not detected.

After four and six weeks increased gallbladder wall thickness under litho+hypo condition was observed (Figure 2A,D). Accordingly, 12.5% and 25% of mice under litho+hypo condition developed cholesterol gallstones. No cholesterol gallstone formation was observed in mice under control, hypo, and litho conditions (Figure 2B,E). Biliary cholesterol monohydrate (ChM) crystal formation was microscopically visible in 28.6% and 50% of mice under litho+hypo conditions at two, four, and six weeks, respectively, whereas neither control, hypo nor litho conditions revealed biliary ChM crystals (Figure 2C,F).

**Figure 3:**
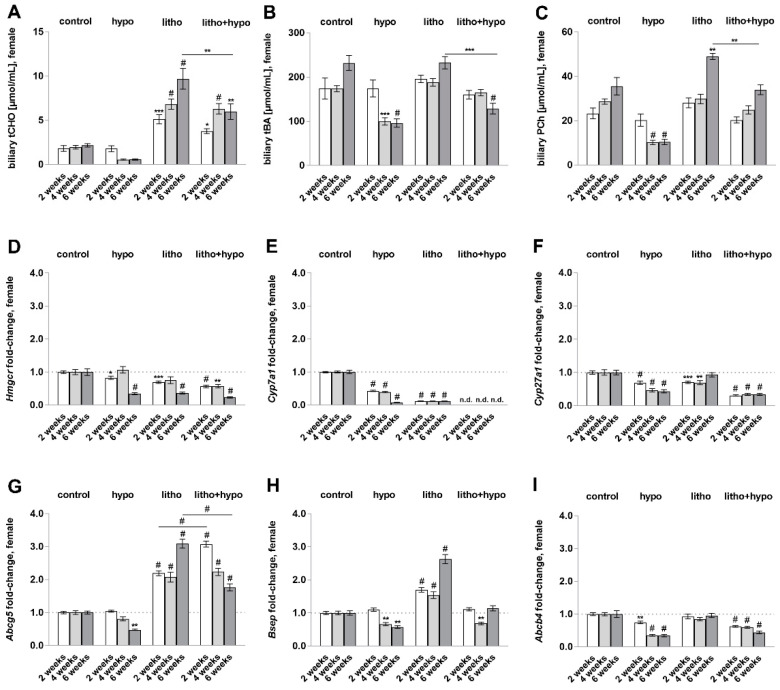
Biliary balance and hepatic expression of genes involved in synthesis and transport of cholesterol, bile acids, and phosphatidylcholine under thyroid hormone deficiency in female C57BL/6J mice. (**A**) Increased total cholesterol concentrations [µmol/mL] under litho and litho+hypo conditions. (**B**) Decreased total bile acid concentrations [µmol/mL] under hypo (4, 6 weeks) and litho+hypo (6 weeks) conditions. (**C**) Decreased phosphatidylcholine concentrations [µmol/mL] under hypo (4, 6 weeks) and increased under litho (6 weeks) conditions. (**D**–**I**) mRNA expression (fold-change) of hepatic cholesterol synthesis enzyme 3-hydroxy-3-methylglutaryl-CoA reductase (*Hmgcr*), bile acid synthesis enzymes cytochrome P450 7A1/27a1 (*Cyp7a1/Cyp27a1*), canalicular cholesterol transporter ATP-binding cassette sub-family G member 5 (*Abcg5*), canalicular bile acid transporter bile salt export pump (*Bsep*), canalicular phospholipid transporter multidrug resistance protein 3 (*Abcb4*) under hypo, litho and litho+hypo relative to control (dotted line: fold-change 1) conditions by quantitative RT-PCR. *18S* (18S ribosomal RNA), *Ppia* (peptidylprolyl isomerase A, cyclophilin A), and *Rpl13 a* (ribosomal protein L13a) were used as reference genes, efficiency corrected Ct method. Data are represented as mean ± SEM, one-way ANOVA followed by Bonferroni post hoc analysis, * *p* < 0.05, ** *p* < 0.01, *** *p* < 0.001, # *p* < 0.0001; n = 3–8/condition.

Biliary cholesterol supersaturation accompanied by a biliary imbalance of cholesterol, bile acids, and phospholipids is thought to be the main cause of cholesterol gallstone formation [13]. Therefore, we investigated biliary concentrations of cholesterol, bile acids, and phosphatidylcholine, the main phospholipid, as well as the hepatic gene expression of the respective synthesis enzymes and canalicular transporters (Figure 3). Biliary total cholesterol concentration was increased in litho and litho+hypo conditions (Figure 3A). In addition, the litho+hypo condition resulted in reduced total cholesterol, total bile acid, and phosphatidylcholine concentrations after six weeks of treatment as compared to litho condition (Figure 3A–C). Hepatic gene expression of *Hmgcr*, *Cyp7a1,* and *Cyp27a1*, enzymes involved in cholesterol and bile acid synthesis, was downregulated in hypo, litho, and litho+hypo conditions (Figure 3D–F). Transcript levels of canalicular cholesterol transporter *Abcg5* were increased in litho and litho+hypo conditions (Figure 3G), whereas canalicular bile acid transporter *Bsep* expression was elevated under litho conditions (Figure 3H). In contrast, phospholipid transporter *Abcb4* was downregulated in the litho+hypo condition (Figure 3I). Under the hypo condition, reduced hepatic *Abcg5*, *Bsep,* and *Abcb4* expression were observed (Figure 3G–I).

### 2.3. TH Deficiency Diminishes Bile Acid Hydrophilicity in Lithogenic Diet Supplemented Female C57BL/6J Mice

**Figure 4:**
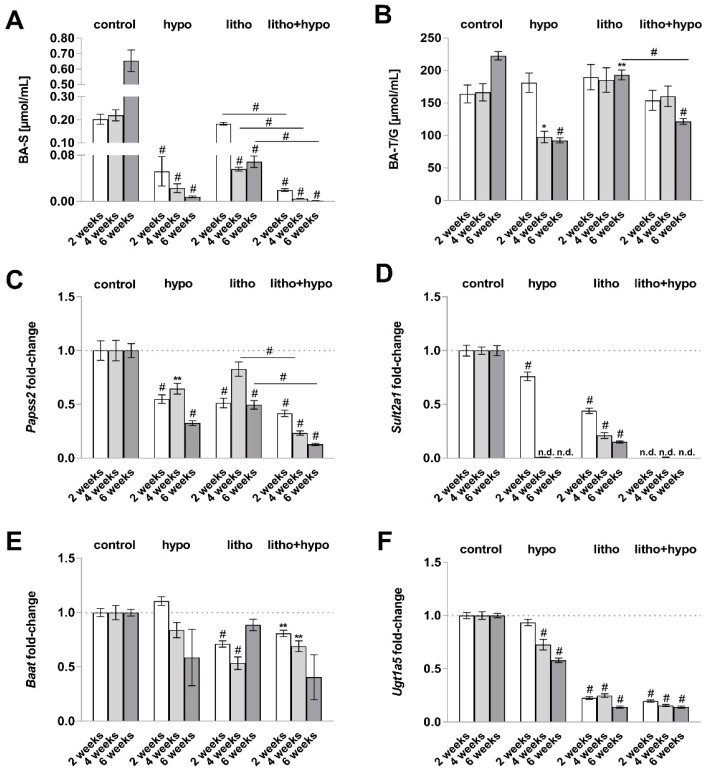
Decreased biliary bile acid conjugates and downregulated gene expression of hepatic detoxification phase II enzymes under thyroid hormone (TH) deficiency in female C57BL/6J mice. (**A**) Bile acid sulfate (BA-S) concentrations [µmol/mL] are reduced under hypo, litho, and litho+hypo conditions. Significant reduction of BA-S after 2, 4, and 6 weeks under litho+hypo as compared to litho condition. (**B**) Glycine- and taurine-conjugated bile acid (BA-T/G) concentrations [μmol/mL] are reduced after 4 and 6 weeks under hypo condition and after 6 weeks under litho+hypo condition as compared to control condition, n = 4–8/condition. (**C**–**F**) mRNA expression (fold-change) of 3′-phosphoadenosine 5′-phosphosulfate synthase 2 (*Papss2*), sulfotransferase family 2a1 (*Sult2a1*), bile acid-CoA:amino acid N-acyltransferase (*Baat*), UDP glucuronosyltransferase family 1 member a5 (*Ugt1a5*) under hypo, litho and litho+hypo relative to control (dotted line: fold-change 1) conditions by quantitative RT-PCR. (**C**) mRNA expression of *Papss2* is decreased in TH-deficient and lithogenic mice. Significant reduction of *Papss2* after 4 and 6 weeks under litho+hypo as compared to litho condition. (**D**) mRNA expression of *Sult2a1* is decreased in TH-deficient and lithogenic mice. (**E**) Gene expression of *Baat* is decreased under litho and litho+hypo conditions. (**F**) Decreased mRNA expression of *Ugt1a5* under hypo, litho and litho+hypo conditions. *18S* (18S ribosomal RNA), *Ppia* (peptidylprolyl isomerase A, cyclophilin A), and *Rpl13 a* (ribosomal protein L13a) were used as reference genes, efficiency corrected Ct method, n = 6–8/condition. Data are represented as mean ± SEM, one-way ANOVA followed by Bonferroni post hoc analysis, * *p* < 0.05, ** *p* < 0.01, # *p* < 0.0001, n.d.: not detected.

In addition to a biliary imbalance, diminished bile acid hydrophilicity may contribute to cholesterol gallstone formation [13]. The ratio of hydrophobic vs. hydrophilic bile acids revealed a decreased amount of hydrophilic primary bile acids under hypo and litho+hypo conditions compared to the control condition (Supplementary Figure S2). Bile acid solubility is facilitated by the conjugation of primary bile acids via hepatic detoxification phase II processes. Further analysis revealed diminished bile acid sulfate concentrations in hypo, litho and litho+hypo conditions, which was even more pronounced in litho+hypo as compared to litho conditions (Figure 4A). In addition, concentrations of glycine- and taurine-conjugated bile acids were reduced after six weeks of treatment under hypo, litho, and litho+hypo conditions (Figure 4B). The expression of *Papss2* (sulfonation), *Sult2a1* (sulfonation), *Baat* (amidation), and *Ugt1b5* (glucuronidation) was diminished either in hypo and/or litho condition (Figure 4C–F).

### 2.4. TH Deficiency Reduces Hepatic Total Cholesterol Content in Lithogenic Diet Supplemented Female but Not Male C57BL/6J Mice

**Figure 5:**
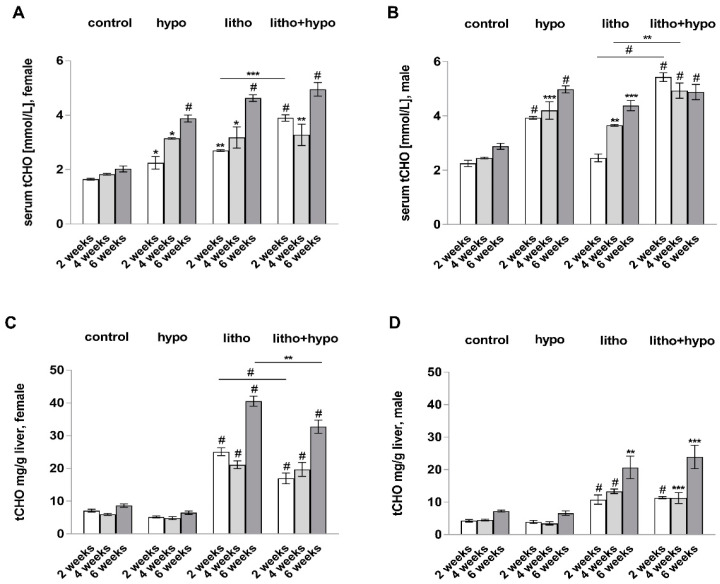
Thyroid hormone deficiency reduces hepatic cholesterol content in lithogenic diet-supplemented female but not male C57BL/6J mice. (**A**,**B**) Serum total cholesterol concentrations [mmol/L] in female and male mice with increased concentrations under hypo, litho, and litho+hypo conditions. (**C**,**D**) Hepatic total cholesterol contents [mg/g liver] in female and male mice. Increase in hepatic cholesterol content under litho and litho+hypo conditions. Reduced cholesterol content in litho+hypo as compared to litho condition in females. Data are represented as mean ± SEM, one-way ANOVA followed by Bonferroni post hoc analysis, * *p* < 0.05, ** *p* < 0.01, *** *p* < 0.001, # *p* < 0.0001, n.d.: not detected, n = 4–8/condition.

As TH deficiency decreases the hydrophilic character of biliary primary bile acids due to a diminished expression of hepatic detoxification phase II enzymes sex-independently, the question arises why female mice revealed a lower cholesterol gallstone prevalence than males. Therefore, serum and hepatic total cholesterol concentrations were investigated. Serum total cholesterol concentrations increased under hypo, litho, and litho+hypo conditions in male and female mice as compared to controls (Figure 5A,B). Hepatic total cholesterol content increased under litho and litho+hypo conditions in both sexes as compared to controls (Figure 5C,D). Female mice showed a higher hepatic cholesterol content as compared to males independent of the treatment regimen (Figure 5C,D). Hepatic cholesterol content was lower in litho+hypo compared to the litho only condition in female mice (Figure 5C).

### 2.5. Hepatobiliary Aquaporin Expression and Localization Sex-Dependently Differ by TH Deficiency in C57BL/6J Mice

**Figure 6:**
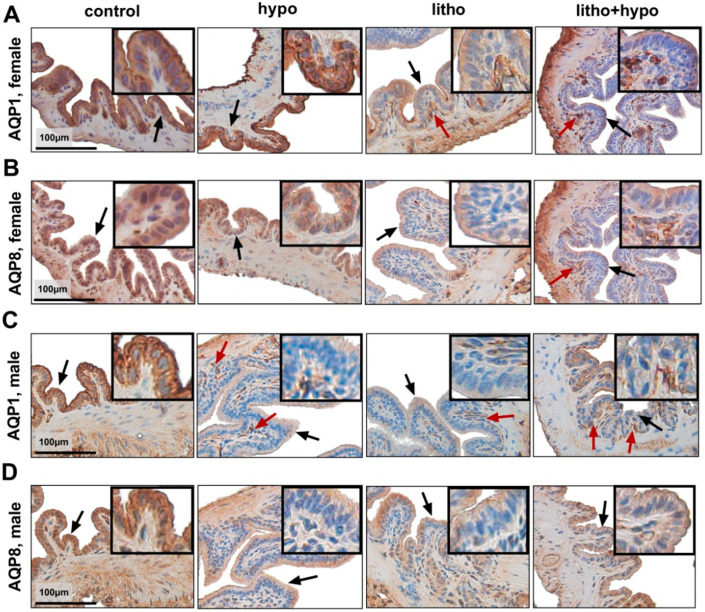
Gallbladder aquaporin expression and localization differ sex-dependently by thyroid hormone deficiency in C57BL/6J mice. Representative immunohistochemistry of aquaporin 1 (AQP1) and aquaporin 8 (AQP8) in gallbladder. (**A**,**B**) Diminished epithelial AQP1 and AQP8 expression (black arrows) and translocation of AQP1 from epithelial cells to subapical vesicles (red arrows) under litho and litho+hypo conditions. (**C**,**D**) Diminished epithelial AQP1 and AQP8 expression (black arrows) and translocation of AQP1 from epithelial cells to subapical vesicles (red arrows) under hypo, litho, and litho+hypo conditions. Scale bar = 100 μm, n = 4/condition.

The gallbladder rearranges bile content by transepithelial water transport processes mediated by aquaporins [14]. Thus, the expression and localization pattern of aquaporin-1 (AQP1) and aquaporin-8 (AQP8) on gallbladder epithelial cells were investigated by immunohistochemistry (Figure 6). In female mice, litho and litho+hypo conditions resulted in the translocation of AQP1 from epithelial cells to subcellular vesicles (Figure 6A), whereas in males a translocation of AQP1 from epithelial cells to subcellular vesicles was observed under hypo, litho and litho+hypo conditions (Figure 6C). A diminished expression of AQP8 was observed under litho and litho+hypo conditions in females (Figure 6B), whereas in males, hypo, litho, and litho+hypo conditions reduced AQP8 expression (Figure 6D). In the liver, the hypo condition reduced *Aqp9* expression and the litho condition increased *Aqp8* expression only in female but not male C57BL/6J mice (Supplementary Figure S3).

## 3. Discussion

Although epidemiological studies demonstrated that women are more prone to develop either hypothyroidism or cholesterol gallstones than men [10,15], a retrospective study reported a predominantly male gallstone prevalence under hypothyroidism [2]. In contrast, in C57BL/6J mice a less cholesterol gallstone susceptibility in females compared to males without manipulation of the TH status has been described [11]. Previously, it was shown that TH deficiency increased the cholesterol gallstone prevalence in male C57BL/6J mice under a lithogenic diet [12]. This was associated with an elevated hydrophobicity of primary bile acids due to diminished gene expression of hepatic detoxification phase II enzymes. Therefore, we conducted a study to investigate the impact of TH deficiency on cholesterol gallstone formation in female C57BL/6J mice. In our study, TH deficiency led to the formation of biliary ChM crystals and cholesterol gallstones in female mice under a lithogenic diet, whereas no biliary ChM crystals or macroscopic cholesterol gallstones were observed in the lithogenic diet only treated animals (Figure 2). In addition, TH deficiency was associated with diminished bile acid hydrophilicity due to decreased gene expression of hepatic detoxification enzymes (Figure 4 and Supplementary Figure S2). These findings strongly correlate with the presence of ChM crystals and cholesterol gallstones in the bile of TH-deficient female mice and reflect the same pathogenic link as recently observed in male C57BL/6J mice [12].

Interestingly, a lithogenic diet reduced hepatic *Dio1* expression and increased TT4 serum concentration in female C57BL/6J mice (Supplementary Table S1) suggesting a local hepatic TH deficient state in contrast to a systemic hyperthyroid condition. Cholesterol-rich diet impacts systemic TH levels and hepatic deiodinases [16,17,18]. However, the well-established low iodine/MMI/perchlorate treatment regimen diminished hepatic *Dio1* expression and serum TT4 concentration under litho+hypo as compared to the litho condition.

Both the low iodine diet and lithogenic diet increased serum total cholesterol concentrations in male and female C57BL/6J mice, whereas hepatic gene expression of enzymes involved in cholesterol synthesis (*Hmgcr*) and degradation (*Cyp7a1*, *Cyp27a1*) were diminished (Figure 3). Hepatic total cholesterol content increased only in lithogenic diet-supplemented animals (Figure 5). It could be speculated that decreased hepatic gene expression under the litho condition is a protective mechanism of the liver due to high dietary cholesterol uptake. In addition, it is well known that TH deficiency diminishes hepatic cholesterol uptake, synthesis, and degradation leading to increased systemic total cholesterol levels in contrast to maintained hepatic total cholesterol [6].

As TH deficiency impacts bile acid hydrophilicity contributing to cholesterol gallstone formation sex-independently, the question arises why female mice revealed a lower cholesterol gallstone prevalence than male mice. In general, our findings are in agreement with the study of Alexander et al. showing that female C57BL/6J mice are less susceptible to cholesterol gallstone formation under a lithogenic diet than males [11]. Authors speculated that increased conversion of cholesterol to bile acids and to esterified cholesterol may explain the lower cholesterol gallstone prevalence in female mice. In our study, female mice showed a higher hepatic cholesterol content as compared to males (Figure 5). Moreover, hepatic cholesterol content was lower in litho+hypo compared to litho only and control conditions in female but not male mice which could contribute to ChM crystal and cholesterol gallstone formation in the bile of female mice under litho+hypo condition. However, gallstone prevalence was much lower in females as compared to males and therefore differences in the hepatic cholesterol content cannot explain the sex-dependent cholesterol gallstone prevalence in TH-deficient C57BL/6J mice.

In murine models of cholestasis, the downregulation of aquaporins in gallbladder epithelial cells was observed [19,20]. In AQP1 knockout (ko) mice water permeability of the gallbladder was approx. 10-fold reduced as compared to wild-type mice [21]. However, bile acid osmolality and biliary bile acid concentration did not differ between AQP1 ko and wild-type mice, arguing against an important function of AQP1 in the pathogenesis of cholestasis. In brain endothelial cells TH influences AQP expression [22] but so far, no data are available for TH’s impact on AQP expression in gallbladder epithelial cells. The present findings of a diminished expression of AQP8 and translocation of AQP1 from epithelial cells to subcellular vesicles, observed in gallbladders of male mice under hypo condition (Figure 6) could contribute to the higher cholesterol gallstone prevalence under TH deficiency in males as compared to females. The drop in aquaporin expression and translocation from epithelial cells to subcellular vesicles may result in reduced biliary water content, increased biliary solute concentrations, and diminished biliary flow thereby promoting cholesterol gallstone formation—a hypothesis that needs to be validated in further studies. The translocation of AQP1 and thereby the removal of AQP1 from the plasma membrane of gallbladder epithelial cells could be a reversible process and translocation from the subapical vesicles back to the plasma membrane upon TH stimulus is conceivable and should be clarified in further studies.

In addition, intestinal factors, e.g., re-absorption of cholesterol and bile acids, intestinal motility, or gut microbiota could contribute to sex- and TH-dependent cholesterol gallstone prevalence. Thus, whether further cellular processes in the hepatobiliary tract may impact cholesterol gallstone prevalence in C57BL/6J mice needs to be investigated.

Moreover, metabolic disorders, e.g., non-alcoholic fatty liver disease (NAFLD), steatohepatitis (NASH), and type-2-diabetes mellitus are associated with intestinal dysmotility, hypertriglyceridemia and/or hyperglycemia and are described to be independent risk factors of gallstone disease [23,24,25,26]. In addition, cholestatic diseases are associated with altered expression of hepatic AQP1, AQP8, and AQP9 [27,28,29]. There is evidence of a lower hepatic AQP9 expression and glycerol permeability in females compared to males potentially contributing to a less severity of females in metabolic disorders [24,25,26]. Here, a low iodine diet reduced hepatic *Aqp9* expression, and a lithogenic diet increased hepatic *Aqp8* expression only in female but not male C57BL/6J mice (Supplementary Figure S3) which could protect the livers of female mice to hepato-steatosis and cholestasis. If and how these sex-specific differences contribute to the lower cholesterol gallstone prevalence in female C57BL/6J mice has to be investigated in further studies. 

In conclusion, we demonstrate that TH deficiency leads to cholesterol gallstone formation due to decreased hydrophilicity of primary bile acids in female C57BL/6J mice under a lithogenic diet. Moreover, the lower cholesterol gallstone prevalence in female compared to male mice under TH deficiency could be caused by sex-dependent differences in the expression and localization of hepatobiliary aquaporins under hypo and litho conditions. Thus, it will be of interest whether the hepatobiliary system may benefit from the modulation of the hepatic TH status and whether, e.g., a local cell-type-specific TH modulation could impact cholestatic liver diseases. 

## 4. Materials and Methods

### 4.1. Animals and Treatment

Three-month-old male and female wild-type C57BL/6JRj mice (Janvier Labs, Le Genest-Saint-Isle, France) were housed under temperature- (23 ± 1 °C) and light-controlled (12:12 h light–dark cycle) conditions. After acclimatization for one week on standard laboratory chow, mice were randomly assigned into four groups. Euthyroid group (control), TH deficient group (hypo), lithogenic group (litho), and lithogenic and TH deficient group (litho+hypo) as described previously [12]. Briefly, TH deficiency (hypo) was induced by a low-iodine diet (MD.1571-95007, ENVIGO, Venray, Netherland) and drinking water supplemented with 0.04% 2-mercapto-1-methylimidazole (MMI), 0.05% sodium perchlorate (ClO4−) and 0.3% saccharine. Cholesterol gallstone formation was either induced by a lithogenic diet (MD.1574, ENVIGO, Venray, Netherland) containing 15.5% fat, 1.25% cholesterol, and 0.5% cholic acid and control drinking water (litho) or a low-iodine lithogenic diet (MD.1573, ENVIGO, Venray, Netherland) containing 15.5% fat, 1.25% cholesterol, and 0.5% cholic acid and drinking water supplemented with 0.04% MMI, 0.05% ClO4−, and 0.3% saccharine (litho+hypo). Mice were treated for two, four, and six weeks (n = 8). Food and water were provided ad libitum.

### 4.2. Collection of Serum, Bile Fluid, and Tissue Samples

At the end of the experiment and after 12 h fasting, mice were euthanized as described previously [30]. Blood samples were harvested by right ventricular heart puncture from deeply anesthetized animals, centrifuged at maximum speed for 20 min at 4 °C and serum samples were stored at −80 °C. Determination of cholesterol gallstones was performed by examination of intact gallbladders under light (n = 6–8). Biliary ChM crystals were investigated by light polarization microscopy using Olympus BX51 upright microscope (Olympus, Hamburg, Germany) (n = 4–8). Bile fluid was collected by puncture with an insulin syringe (BD Micro-fine, 29G, Heidelberg, Germany) and stored at −80 °C. Tissues were isolated from liver heparinized saline perfused mice and processed as follows: liver tissue was snap frozen in liquid nitrogen and stored at −80 °C or liver tissue was embedded in Tissue-Tek O.C.T. compound (Plano GmbH, Wetzlar, Germany), snap frozen in liquid nitrogen and stored at −80 °C. For histological analysis, liver and gallbladder tissues were stored in 4% paraformaldehyde in 1× PBS at room temperature.

### 4.3. Serum Measurements

TT4 serum concentration (n = 6–8) was measured using an ELISA kit according to the manufacturer’s instructions (EIA 1781, DRG Instruments GmbH, Marburg, Germany) with an intra-assay coefficient of variation of 3.1–4.3% and an inter-assay coefficient of variation of 2.4–4.5%. Serum enzyme activities (n = 4) of alanine transaminase (ALT) and aspartate transaminase (AST) and total cholesterol concentrations (n = 4–8) were analyzed by LABOKLIN GmbH & Co. KG (LABOKLIN, Bad Kissingen, Germany).

### 4.4. Bile Measurements

Biliary bile acid concentrations (n = 4–8) were determined by negative electrospray (ESI) liquid chromatography–tandem mass spectrometry (LC-MS/MS) in multiple reaction monitoring (MRM) mode on an Agilent 6460 triple quadrupole mass spectrometer (Agilent, Waldbronn, Germany) coupled to an Agilent 1290 HPLC system [31]. Briefly, 5 μL of diluted bile (1:100) was spiked with 20 μL of internal standard solution in methanol:water (1:1 *v*/*v*). Protein precipitation was performed with 30 μL of methanol, followed by centrifugation. The supernatant was used for LC-MS/MS analysis (Supplementary Table S2). Cholesterol concentrations (n = 4–8) in bile were determined by gas chromatography–mass spectrometry (GC-MS) using 10 μL of diluted bile (1:100) [32]. Biliary phosphatidylcholine concentrations (n = 3–8) were measured using a colorimetric coupled enzyme assay (MAK049, Sigma-Aldrich, St. Louis, MO, USA) according to the manufacturer’s instructions.

### 4.5. Hepatic Total Cholesterol Content

Ten mg liver tissue was homogenized in 200 μL of chloroform:isopropanol:NP40 solution (7:11:0.1 *v*/*v*/*v*) using Tissue Lyser II (frequency 22/s, 5 min) and stainless steel beads (5 mm) (both Qiagen, Hilden, Germany). After centrifugation for 3 min at 500× *g* and 4 °C, samples were homogenized. After second centrifugation step for 3 min at 9.500× *g* and 4 °C, the supernatant was collected and lyophilized using Speed Vac^®^ for 1 h at 35 °C. The lyophilisate was diluted in 50 μL cholesterol assay buffer and processed further in the Cholesterol Quantitation Kit (MAK043, Sigma-Aldrich, St. Louis, MO, USA) according to manufacturer’s instructions.

### 4.6. Histological Analysis

Liver and gallbladder samples were fixed in 4% paraformaldehyde in 1× PBS, embedded in paraffin, and 4 μm thick sections were used for hematoxylin and eosin (HE) staining. Gallbladder wall thickness was quantified using Image J software (1.52a, NIH, Madison, WI, USA). For each gallbladder (n = 3–4/condition), wall thickness is the mean value of 6 determinations in the fundus area (10× magnification). To determine neutral lipids, Oil Red O staining of frozen sections of TissueTek-embedded liver samples (5 μm) was performed and quantified using Adobe Photoshop CC2017 as follows: Lipid intensities were determined by isolation of the neutral lipid channel from the photomicrographs followed by evaluation of the mean density values using the histogram feature of five fields of views per image (60× magnification, n = 4). HE staining was digitalized by Aperio scanner (Leica, Mannheim, Germany) and Oil Red O staining was viewed on the Olympus BX51 upright microscope (Olympus, Hamburg, Germany).

### 4.7. Immunohistochemistry

Gallbladder tissues were fixed in 4% paraformaldehyde in 1× PBS, embedded in paraffin, and 4 μm thick sections were used. Sections were incubated with Neo-Clear^®^ (3 × 5 min) and rehydrated using descending EtOH concentrations (100%, 96%, 70%) and H_2_O_milli_ for 3 min each. The sections were incubated in 1× citrate buffer (pH = 6) for 20 min at 95 °C, washed 2 × 2 min with 1× Zytomed Systems wash buffer, incubated for 5 min with 3% hydrogen peroxide, washed 3 × 5 min with 1× Zytomed Systems wash buffer, incubated for 40 min with blocking solution, washed 1 × 2 min with 1× Zytomed Systems wash buffer and incubated with primary antibody (rabbit anti-AQP1, ab2219, Millipore, Darmstadt, Germany, 1:2000 and rabbit anti-AQP8, ABIN872611, antibodies-online, Aachen, Germany, 1:2000) at 4 °C overnight. Then, sections were incubated with PostBlock for 20 min followed by 30 min incubation with PolyHRP and 10 min incubation with ZytoDAB. Nuclei were stained with hematoxylin (1:5 in H_2_O_milli_) for 1 min at room temperature. After washing with H_2_O_milli_ the sections were dehydrated using ascending EtOH concentrations (70%, 96%, and 100%) as well as Neo-Clear^®^ (3 × 5 min). A secondary antibody only control was used as negative control. Finally, sections were covered with Neo-Mount^®^ and coverslips and visualized with Olympus BX51 Upright microscope (Olympus, Hamburg, Germany).

### 4.8. Quantitative Real-Time PCR

RNA was isolated using RNeasy Kit (Qiagen, Hilden, Germany) and reverse transcribed into cDNA (Life Technologies, Darmstadt, Germany). Quantitative real-time PCR (qRT-PCR) was performed as described previously [30]. Sequences of oligonucleotides designed using PrimerBlast (NCBI, Bethesda, MD, USA) and synthesized by Eurofins (Eurofins MWG, Munich, Germany) are provided in Supplementary Table S3. Relative expression of genes was normalized to the control group (n = 6–8).

### 4.9. Statistics

All data are shown as mean ± SEM with n = 6–8 (HE staining liver, wet liver weight, gallstone prevalence, hepatic gene expression, serum TT4), n = 4–8 (biliary ChM crystals, serum, bile, and hepatic total cholesterol, biliary bile acids), n = 3–8 (biliary phosphatidylcholine), n = 4 (serum transaminases, gallbladder AQP expression) and n = 3–4 (HE staining gallbladder). Statistical analysis was performed using GraphPad Prism 7 software (www.graphpad.com, accessed on 15 January 2020). One-way ANOVA followed by Bonferroni post hoc analysis was used. Expression data of qRT-PCR were analyzed using anti-logarithmic data. Values of * *p* < 0.05, ** *p* < 0.01, *** *p* < 0.001 and # *p* < 0.0001 were considered statistically significant.

## Data Availability

Not applicable.

## Supplementary Materials

The following supporting information can be downloaded at: https://www.mdpi.com/article/10.3390/ijms232012355/s1.
