# Supplementary material for: Hepatobiliary Thyroid Hormone Deficiency Impacts Bile Acid Hydrophilicity and Aquaporins in Cholestatic C57BL/6J Mice
Source: Int J Mol Sci. 2022 Oct 15;23(20):12355. doi: 10.3390/ijms232012355 (PMC9603918; doi:10.3390/ijms232012355)
Supplement: Supplementary file 1 [file ijms-23-12355-s001.zip › Kube et al_Supplementary Legends S1-S3_IJMS_R.pdf]

Supplementary Figure S1. Serological liver function tests in female C57BL/6J mice. (**A**) Alanine transaminase (ALT) activity [U/L] is increased after 2 weeks under litho and litho+hypo conditions as compared to control. (**B**) Aspartate transaminase (AST) activity [U/L] is increased after 2 weeks under litho+hypo condition. Data are represented as mean±SEM, one-way ANOVA followed by Bonferroni post hoc analysis, #p<0.0001; n=4/condition.

Supplementary Figure S2. Decreased amount of hydrophilic primary bile acids in bile under thyroid hormone deficiency in female C57BL/6J mice. (**A**) Decreased hydrophilicity (%) of total bile acids in bile under hypo, litho and litho+hypo conditions. (**B**) Decreased hydrophilicity (%) of biliary primary bile acids under hypo, litho and litho+hypo conditions. (**C**) Decreased hydrophilicity (%) of biliary secondary bile acids under litho and litho+hypo conditions. n=4-8/condition.

Supplementary Figure S3. Hepatic aquaporin gene expression under thyroid hormone deficient and lithogenic condition in female and male C57BL/6J mice. (A-F) mRNA expression (fold-change) of hepatic aquaporin 1 (*Aqp1*), aquaporin 8 (*Aqp8*) and aquaporin 9 (*Aqp9*) under hypo, litho and litho+hypo relative to control (dotted line: fold-change 1) conditions by quantitative RT-PCR. Either *18S* (18S ribosomal RNA), *Ppia* (peptidylprolyl isomerase A, cyclophilin A) and *Rpl13 a* (ribosomal protein L13a) or *18S* and *Ppia* were used as reference genes, efficiency corrected Ct method. Data are represented as mean±SEM, one-way ANOVA followed by Bonferroni *post hoc* analysis, *p<0.05, **p<0.01, ***p<0.001, #p<0.0001; n=6-8/condition.
