# Supplementary material for: Hepatobiliary Thyroid Hormone Deficiency Impacts Bile Acid Hydrophilicity and Aquaporins in Cholestatic C57BL/6J Mice
Source: Int J Mol Sci. 2022 Oct 15;23(20):12355. doi: 10.3390/ijms232012355 (PMC9603918; doi:10.3390/ijms232012355)
Supplement: Supplementary file 1 [file ijms-23-12355-s001.zip › Kube et al_Supplementary Tables S1-S3_IJMS_R.pdf]

Supplementary Table S1. Serum total thyroxine (TT4) concentrations [µg/dL]. Detection limit of 0.5 µg/dL. mRNA expression (fold-change) of hepatic deiodinase 1 (*Dio1*) and thyroxine-binding globulin (*Tbg)* under hypo, litho and litho+hypo relative to control conditions by quantitative RT-PCR. *18S* (18S ribosomal RNA), *Ppia* (peptidylprolyl isomerase A, cyclophilin A) and *Rpl13 a* (ribosomal protein L13a) were used as reference genes, efficiency corrected Ct method. Data are presented as mean±SEM, one-way ANOVA followed by Bonferroni *post hoc* analysis, ***p*<0.01, ****p*<0.001 #*p*<0.0001; n.d.: not detected; §data from Kube et al.; https://doi.org/10.1055/a-1404-7939; n=6-8/condition.

**Parameter**	**Time (weeks)**	**Control**	**Hypo**	**Litho**	**Litho+Hypo**
**TT4 ± SEM [µg/dL]**	2	3.63±0.29	1.45±0.40**	6.21±0.57***	0.89±0.04***
	4	2.27±0.26	0.18±0.01#	4.56±0.09#	0.50±0.04#
	6§	3.63±0.24	0.28±0.05#	5.03±0.24#	0.41±0.05#
**hepatic *Dio1* ± SEM (fold-change)**	2	1.00±0.12	n.d.	0.38±0.04#	n.d.
	4	1.00±0.05	n.d.	0.36±0.03#	n.d.
	6§	1.00±0.04	n.d.	0.34±0.03#	n.d.
**hepatic *Tbg* ± SEM (fold-change)**	2	1.00±0.13	2.16±0.19	1.98±0.21	8.99±0.77#
	4	1.00±0.07	4.97±0.38#	1.63±0.12	5.48±0.31#
	6§	1.00±0.08	8.04±0.70#	1.09±0.11	9.32±0.94#

Supplementary Table S2. Bile acid derivates in bile of 2, 4 and 6 weeks treated female C57BL/6J mice. CA – cholic acid, CDCA - chenodeoxycholic acid, DCA – deoxycholic acid, LCA – lithocholic acid, MCAα/β/σ – murine cholic acid isoforms α/β/σ, UDCA – ursodeoxycholic acid, T and G prefixes denotes glycine and taurine conjugation. Data are represented as mean in µmol/mL ± SEM. ULOD: under lower limit of detection; n=4-8/condition.

Primary BAs	BA derivate	Time	Control [µmol/mL]	Hypo [µmol/mL]	Litho [µmol/mL]	Litho+Hypo [µmol/mL]
hydrophilic	TCA-S	2 wks	0.15±0.02	0.14±0.04	0.04±0.003	0.01±0.003
		4 wks	0.17±0.01	0.02±0.002	0.05±0.004	0.003±0.001
		6 wks	0.56±0.07	0.007±0.0009	0.06±0.01	0.001±0.0001
	CA-S	2 wks	0.05±0.02	0.04±0.009	0.01±0.002	0.005±0.001
		4 wks	0.05±0.003	0.003±0.0002	0.008±0.001	0.001±0.0005
		6 wks	0.09±0.02	0.001±0.0006	0.008±0.002	ULOD
	TMCA	2 wks	93.31±10.71	75.21±11.07	13.41±1.44	2.91±0.18
		4 wks	89.24±4.62	27.23±2.96	13.03±2.93	2.59±0.15
		6 wks	130.38±8.31	29.22±2.34	14.25±0.95	3.15±0.16
	αMCA	2 wks	1.53±0.47	0.72±0.14	0.06±0.02	0.03±0.01
		4 wks	1.01±0.15	0.13±0.02	0.05±0.01	0.03±0.008
		6 wks	1.09±0.30	0.37±0.21	0.15±0.06	0.05±0.02
	βMCA	2 wks	1.41±0.43	0.51±0.06	0.12±0.05	0.03±0.01
		4 wks	0.78±0.16	0.07±0.01	0.06±0.01	0.01±0.004
		6 wks	1.72±0.49	0.13±0.06	0.22±0.09	0.03±0.01
	7-DHCA	2 wks	1.55±0.47	0.69±0.13	0.47±0.12	0.37±0.09
		4 wks	1.35±0.23	0.21±0.05	0.25±0.09	0.36±0.12
		6 wks	0.81±0.28	0.15±0.07	0.42±0.14	0.44±0.15
	12-DHCA	2 wks	0.55±0.14	0.19±0.02	0.06±0.01	0.04±0.004
		4 wks	0.51±0.14	0.08±0.02	0.03±0.002	0.03±0.004
		6 wks	0.23±0.05	0.09±0.03	0.05±0.007	0.03±0.008
hydrophobic	GCA	2 wks	0.13±0.03	0.31±0.02	0.57±0.04	0.56±0.08
		4 wks	0.12±0.01	0.18±0.02	1.56±0.89	1.11±0.18
		6 wks	0.24±0.02	0.08±0.006	1.84±0.62	0.94±0.28
	TCA	2 wks	53.74±8.18	77.19±7.88	139.89±7.01	107.93±7.33
		4 wks	56.94±2.84	61.39±5.20	136.38±6.79	109.99±6.05
		6 wks	72.88±4.40	54.98±4.29	142.86±5.18	85.94±4.79
	CA	2 wks	4.19±1.55	4.03±0.48	5.00±1.28	5.44±1.71
		4 wks	2.62±0.33	1.43±0.48	1.87±0.89	3.73±1.17
		6 wks	4.44±1.16	2.45±1.49	5.88±2.28	6.56±3.01
	CDCA	2 wks	0.003±0.001	0.002±0.001	0.0006±0.0002	ULOD
		4 wks	0.001±0.0003	0.0009±0.00003	ULOD	ULOD
		6 wks	0.002±0.0005	0.003±0.001	0.001±0.0004	0.001±0.0005
	TCDCA	2 wks	4.05±0.64	4.02±0.60	0.76±0.05	0.39±0.02
		4 wks	3.78±0.07	3.46±0.59	0.91±0.17	0.47±0.04
		6 wks	4.91±0.41	3.15±0.43	1.15±0.09	0.36±0.03

Secondary BAs	BA derivate	Time	Control [µmol/mL]	Hypo [µmol/mL]	Litho [µmol/mL]	Litho+Hypo [µmol/mL]
hydrophilic	ωMCA	2 wks	0.58±0.15	0.29±0.03	0.04±0.009	0.009±0.002
		4 wks	0.52±0.13	0.04±0.008	0.03±0.007	0.006±0.001
		6 wks	0.54±0.15	0.09±0.03	0.06±0.02	0.03±0.01
	TUDCA	2 wks	6.82±0.92	6.23±0.86	0.83±0.05	0.31±0.01
		4 wks	8.12±0.41	3.01±0.47	0.92±0.19	0.29±0.01
		6 wks	7.37±0.38	2.55±0.15	0.95±0.08	0.24±0.02
	UDCA	2 wks	0.005±0.001	0.003±0.001	ULOD	ULOD
		4 wks	0.004±0.001	0.001±0.0004	ULOD	ULOD
		6 wks	0.004±0.001	0.003±0.001	0.002±0.001	0.003±0.001
	HDCA	2 wks	0.005±0.002	0.002±0.001	ULOD	ULOD
		4 wks	0.006±0.002	ULOD	ULOD	ULOD
		6 wks	0.004±0.001	0.004±0.003	ULOD	ULOD
hydrophobic	TDCA	2 wks	5.92±0.88	4.76±0.91	34.52±5.26	42.13±2.79
		4 wks	8.37±0.47	2.52±0.67	32.79±1.62	46.13±3.29
		6 wks	6.87±0.85	2.65±0.24	32.31±4.23	31.03±3.44
	DCA	2 wks	0.005±0.002	ULOD	0.02±0.006	0.06±0.02
		4 wks	0.004±0.001	ULOD	0.007±0.001	0.04±0.008
		6 wks	0.003±0.001	ULOD	0.04±0.01	0.08±0.04
	TLCA	2 wks	0.09±0.007	0.06±0.006	0.01±0.002	0.01±0.0008
		4 wks	0.11±0.01	0.04±0.02	0.02±0.002	0.02±0.001
		6 wks	0.08±0.009	0.03±0.004	0.02±0.002	0.02±0.002
	LCA	2 wks	ULOD	ULOD	ULOD	ULOD
		4 wks	0.0006±0.00006	ULOD	0.0004±0.0001	ULOD
		6 wks	0.0006±0.0002	ULOD	ULOD	ULOD

Supplementary Table S3. Oligonucleotides used for amplification of genes by quantitative RT-PCR. Oligonucleotides were designed using PrimerBlast (NCBI) and synthesized by Eurofins (Eurofins MWG Synthesis, Ebersberg, Germany).

Gene name	Abbr.	Sequence (5`→3`) For	Sequence (5`→3`) Rev	Product length (bp)	GC (%) For	GC (%) Rev	Tm (°C) For	Tm (°C) Rev
**18S ribosomal RNA**	*18S*	CGG CTA CCA CAT CCA AGG AA	GCT GGA ATT ACC GCG GCT	187	55.0	55.0	59.4	59.3
**Multidrug resistance protein 3**	*Abcb4*	GAT GGA TCT TGA GGC AGC GA	TTC TCT GCC TTG GTT GCT GA	88	55.0	50.0	59.3	57.3
**ATP-binding cassette sub-family G member 5**	*Abcg5*	CTC CGC GGA CTT CTA CAA CA	CAT GAC CTT GGG GTC CTG AA	166	55.0	55.0	59.3	59.3
**Aquaporin 1**	*Aqp1*	CTT GCC ATT GGC TTG TCT GTG G	CCA GTG GTT TGA GAA GGT GCG G	123	54.6	54.6	62.5	62.2
**Aquaporin 8**	*Aqp8*	TAC CTT GGG GAA CAT CAG CG	TCT GGA CTC ACC ACT TTA GCC	159	55.0	52.4	59.3	59.8
**Aquaporin 9**	*Aqp9*	TTG CAA CGG CAG TTG TGA TG	GGC CAT GAG TCC GTC ATA GT	200	50.0	55.0	57.3	59.3
**Bile acid-CoA:amino acid N-acyltransferase**	*Baat*	CCT CAC TTC CAT AGA GGA ATG TCT T	CAC TCT CCA TCC TGT ATT TTC T	85	44.0	61.3	44.0	61.3
**Bile salt export pump**	*Bsep*	ACA TCT GTA GGG TTG TTG AGT GA	CAA AGA AGC CAA CTC GAG CG	182	45.0	55.0	55.3	59.3
**CytochromeP450 Family7 Subfamily A Member 1**	*Cyp7a1*	TTC TGC GAA GGC ATT TGG AC	GAT TTA GGA AGG CCC GGA GG	171	50.0	60.0	57.3	61.4
**CytochromeP450 Family27 Subfamily A Member 1**	*Cyp27a1*	TTT AAG GCA TCC GTG TAG AGC	CCT CAC CTA TGG GAT CTT CAT G	132	47.6	50.0	57.9	60.3
**Deiodinase Type 1**	*Dio1*	GGG CAG GAT CTG CTA CAA GG	CGT GTC TAG GTG GAG TGC AA	98	60.0	55.0	61.4	59.3
**3-Hydroxy-3-methylglutaryl-CoA reductase**	*Hmgcr*	TCG CTG GTC CTA GAG CTT TC	CCA GCG ACT ATG AGC GTG AA	171	55.0	59.4	55.0	59.4
**3-Phosphoadenosine 5-phosphosulfate synthase 2**	*Papss2*	CCC ACC TCA CAG ACC TTC AC	GGT GGA TTT TTG CTG GTC TCT TT	187	60.0	43.5	61.4	58.9
**Peptidylpropyl isomerase A, cyclophilin A**	*Ppia*	CTT GGG CCG CGT CTC CTT CG	GCG TGT AAA GTC ACC CTG GC	160	70.0	60.0	65.5	61.4
**Ribosomal protein L13a**	*Rpl13a*	GGG CAG GTT CTG GTA TTG GA	GGG GTT GGT ATT CAAT CCG CT	162	55.0	52.4	59.3	59.8
**Sulfotransferase family 2A1**	*Sult2a1*	ATC TCC AGC TCA AAG ACC AAT TCT	TCC ATG TTG CTG GGG TTC TG	109	50.0	50.0	57.3	57.3
**Thyroxine binding globuline**	*Tbg*	TGG GCA TGT GCT ATC TTC A	GAG TGG CAT TTT GTT GGG GC	153	47.4	55.0	54.5	59.3
**UDP glucuronosyltransferase family 1 member a5**	*Ugt1a5*	ATG CCC CTA CAA GGA TTA GTG G	AGC ACC TTC TCA CCT TCA GTC	71	50.0	52.4	60.3	59.8
