# Supplementary figures and images for: Hepatobiliary Thyroid Hormone Deficiency Impacts Bile Acid Hydrophilicity and Aquaporins in Cholestatic C57BL/6J Mice
Source: Int J Mol Sci. 2022 Oct 15;23(20):12355. doi: 10.3390/ijms232012355 (PMC9603918; doi:10.3390/ijms232012355)

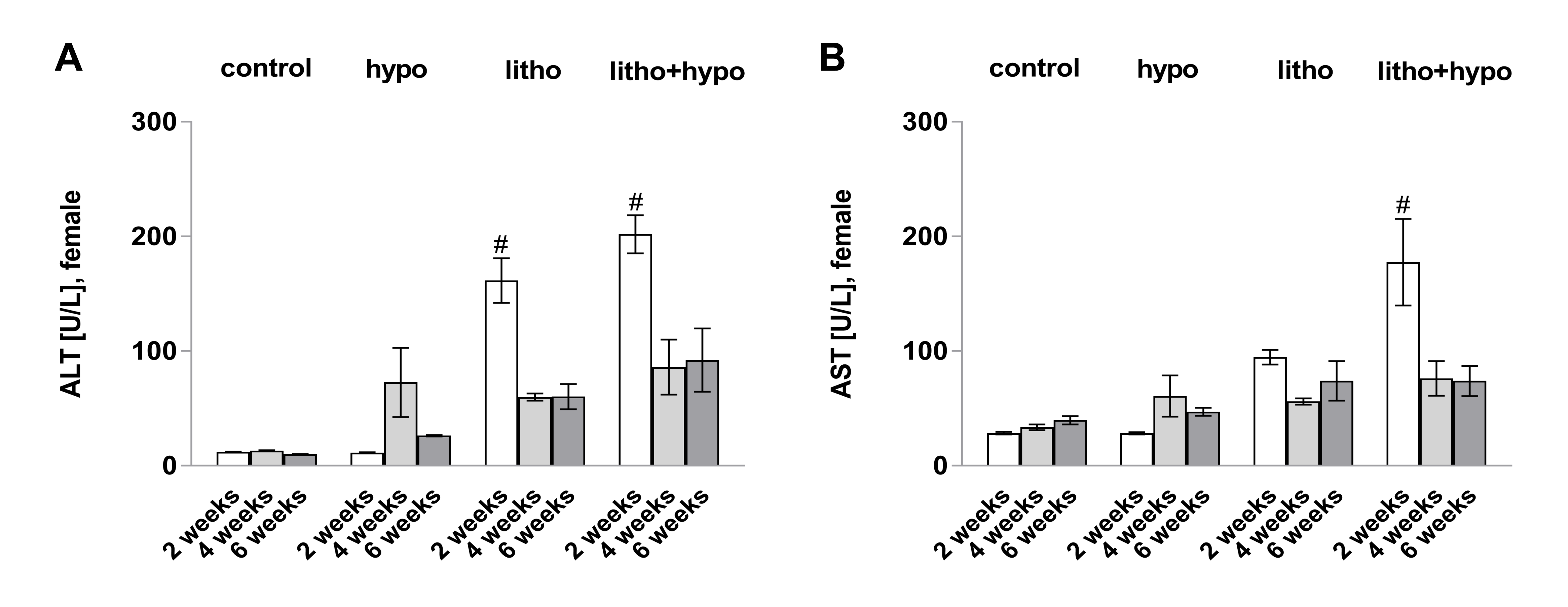

Supplement: Supplementary file 1 [file ijms-23-12355-s001.zip › Kube et al_Supplementary Figure S1.tif]

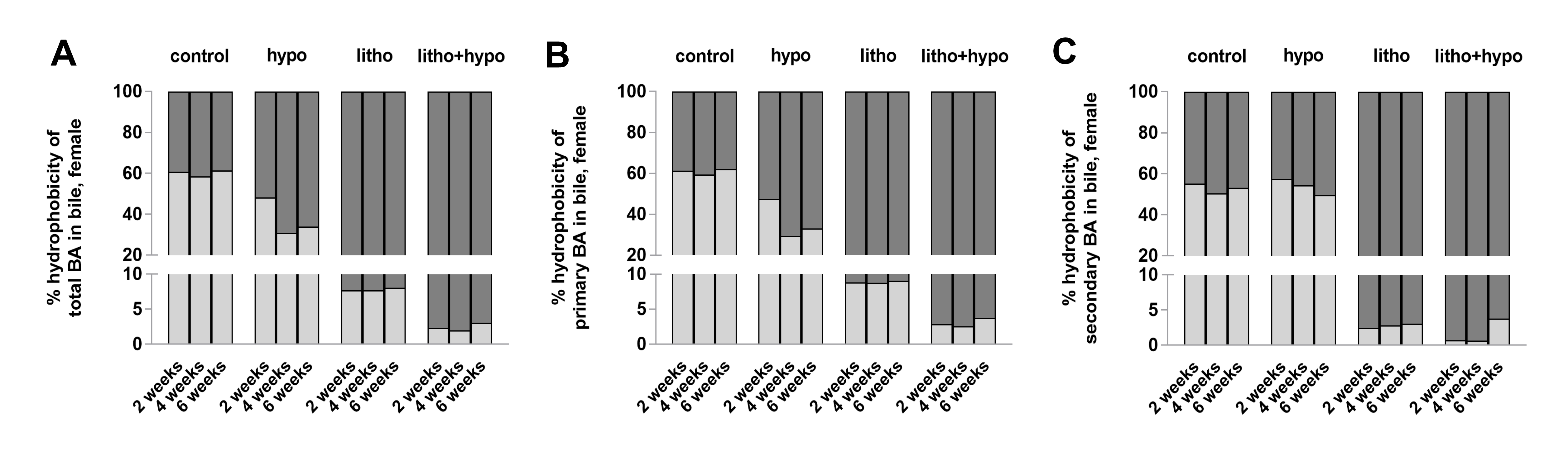

Supplement: Supplementary file 1 [file ijms-23-12355-s001.zip › Kube et al_Supplementary Figure S2.tif]

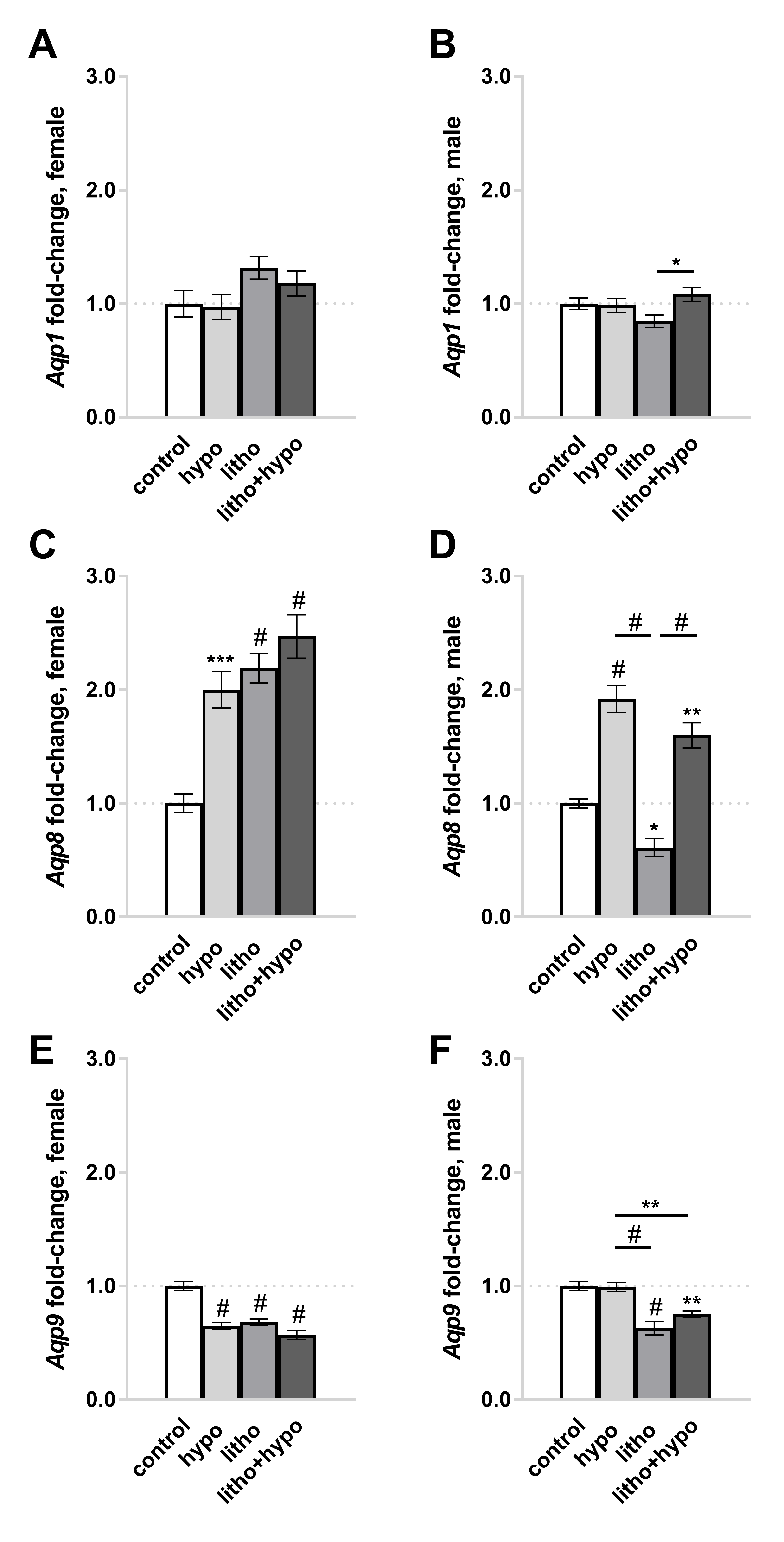

Supplement: Supplementary file 1 [file ijms-23-12355-s001.zip › Kube et al_Supplementary Figure S3.tif]
